# Night Shift Work Affects Urine Metabolite Profiles of Nurses with Early Chronotype

**DOI:** 10.3390/metabo8030045

**Published:** 2018-08-21

**Authors:** Markus Rotter, Stefan Brandmaier, Marcela Covic, Katarzyna Burek, Johannes Hertel, Martina Troll, Erik Bader, Jonathan Adam, Cornelia Prehn, Birgit Rathkolb, Martin Hrabe de Angelis, Hans Jörgen Grabe, Hannelore Daniel, Thomas Kantermann, Volker Harth, Thomas Illig, Dirk Pallapies, Thomas Behrens, Thomas Brüning, Jerzy Adamski, Heiko Lickert, Sylvia Rabstein, Rui Wang-Sattler

**Affiliations:** 1 Research Unit of Molecular Epidemiology, Helmholtz Zentrum München, 85764 München-Neuherberg, Germany; markus.rotter@helmholtz-muenchen.de (M.R.); stefan.brandmaier@helmholtz-muenchen.de (S.B.); marcela.covic@helmholtz-muenchen.de (M.C.); martina.troll@helmholtz-muenchen.de (M.T.); ErikSBader@web.de (E.B.); jonathanadam86@gmail.com (J.A.); 2 Institute of Epidemiology, Helmholtz Zentrum München, 85764 München-Neuherberg, Germany; 3 German Center for Diabetes Research (DZD), 85764 München-Neuherberg, Germany; birgit.rathkolb@helmholtz-muenchen.de (B.R.); hrabe@helmholtz-muenchen.de (M.H.d.A.); adamski@helmholtz-muenchen.de (J.A.); heiko.lickert@helmholtz-muenchen.de (H.L.); 4 Institute for Prevention and Occupational Medicine of the German Social Accident Insurance, Institute of the Ruhr University Bochum (IPA), 44721 Bochum, Germany; burek@ipa-dguv.de (K.B.); pallapies@ipa-dguv.de (D.P.); behrens@ipa-dguv.de (T.Be.); bruening@ipa-dguv.de (T.Br.); rabstein@ipa-dguv.de (S.R.); 5 Department of Psychiatry and Psychotherapy, University Medicine Greifswald, 17489 Greifswald, Germany; hertelj@uni-greifswald.de (J.H.); grabeh@uni-greifswald.de (H.J.G.); 6 Institute Institute of Diabetes and Regeneration Research, Helmholtz Zentrum München, 85764 München-Neuherberg, Germany; 7 Genome Analysis Center, Institute of Experimental Genetics, Helmholtz Zentrum München, German Research Center for Environmental Health, 85764 München-Neuherberg, Germany; prehn@helmholtz-muenchen.de; 8 Chair for Molecular Animal Breeding and Biotechnology, Gene Center and Department of Veterinary Sciences, and Center for Innovative Medical Models (CiMM), LMU Munich, 81377 Munich, Germany; 9 German Mouse Clinic (GMC), Institute of Experimental Genetics, Helmholtz Zentrum München, 85764 München-Neuherberg, Germany; 10 Institute of Experimental Genetics, Helmholtz Zentrum München, 85764 München-Neuherberg, Germany; 11 Chair of Experimental Genetics, Center of Life and Food Sciences Weihenstephan, Technische Universität München, 85353 Freising, Germany; 12 German Center for Neurodegenerative Diseases (DZNE), Rostock/Greifswald, 17489 Greifswald, Germany; 13 Molecular Nutrition Unit, Center of Life and Food Sciences Weihenstephan, Technische Universität München, 85354 Freising, Germany; hannelore.daniel@tum.de; 14 University of Applied Sciences for Economics and Management (FOM), 45127 Essen, Germany; thomas.kantermann@fom.de; 15 SynOpus, 44789 Bochum, Germany; 16 Institute for Occupational Medicine and Maritime Medicine (ZfAM), University Medical Center Hamburg-Eppendorf, 20246 Hamburg, Germany; volker.harth@bgv.hamburg.de; 17 Hannover Unified Biobank, Hannover Medical School, 30625, Hannover, Germany; Illig.Thomas@mh-hannover.de; 18 Institute for Human Genetics, Hannover Medical School, Carl-Neuberg-Strasse 1, D-30625 Hanover, Germany; 19 Institute of Stem Cell Research, Helmholtz Zentrum München, 85764 München-Neuherberg, Germany

**Keywords:** metabolomics, urine normalization, women’s’ health, night shift work, chronotypes

## Abstract

Night shift work can have a serious impact on health. Here, we assess whether and how night shift work influences the metabolite profiles, specifically with respect to different chronotype classes. We have recruited 100 women including 68 nurses working both, day shift and night shifts for up to 5 consecutive days and collected 3640 spontaneous urine samples. About 424 waking-up urine samples were measured using a targeted metabolomics approach. To account for urine dilution, we applied three methods to normalize the metabolite values: creatinine-, osmolality- and regression-based normalization. Based on linear mixed effect models, we found 31 metabolites significantly (false discovery rate <0.05) affected in nurses working in night shifts. One metabolite, acylcarnitine C10:2, was consistently identified with all three normalization methods. We further observed 11 and 4 metabolites significantly associated with night shift in early and late chronotype classes, respectively. Increased levels of medium- and long chain acylcarnitines indicate a strong impairment of the fatty acid oxidation. Our results show that night shift work influences acylcarnitines and BCAAs, particularly in nurses in the early chronotype class. Women with intermediate and late chronotypes appear to be less affected by night shift work.

## 1. Introduction

About one fifth of employees in industrialized countries are working in some type of shift schedule [1]. Shift work was reported to have adverse health effects and increase the chance for diseases like obesity and type 2 diabetes [2,3,4,5]. When forced to work at a non-standard time (e.g., night shift), which is not in concordance with the personal inner clock, individuals tend to develop a “social jet lag,” a discrepancy between sleep timing on work days and free days [2,6]. The difference in the preferred personal timing or chronotype plays a major role in individual sleep duration and health risk when working night shift [5,7]. Previous research showed that female night shift workers with morning preferences had a higher risk for breast cancer compared to those with evening preferences [3]. The underlying metabolic pathways affected by shift work and the role of the individual chronotype in this respect have not been studied in detail.

Targeted metabolomic profiling in human blood has been used to assess lifestyle or disease effects such as pre-diabetes, sleep curtailment and sleep deprivation [8,9,10,11,12,13]. However, no real-life study investigating the impact of night shift work on the urine metabolic profile of participants with early, intermediate and late chronotype has been reported. Major advantages of urine bio samples are their non–invasive sampling, moreover, urine samples are well studied with respect to age, obesity and storage conditions [14,15,16,17]. However, as urine samples are susceptible to a variety of factors such as water intake, it was suggested to either use alternative methods besides creatinine- or osmolality-normalizations, or to apply more than one normalization method [18,19,20].

Here, we have used the Munich Chronotype Questionnaire for shift workers (MCTQ^Shift^) to assess the chronotype of participants working in shift work [21]. We collected about 3640 longitudinal multi-time-point urine samples and analysed 424 waking-up samples (a proxy for fasting samples) from 68 female nurses working on several consecutive days during day or night shift. Targeted urine metabolite profiles were normalized with creatinine-, osmolality- and regression based normalization (RBN) and the results allowed us to investigate the influence of night shift on the targeted metabolite profile in urine of female nurses while considering their individual chronotypes.

## 2. Results

### 2.1. Characteristics of Participants

**Figure 1:**
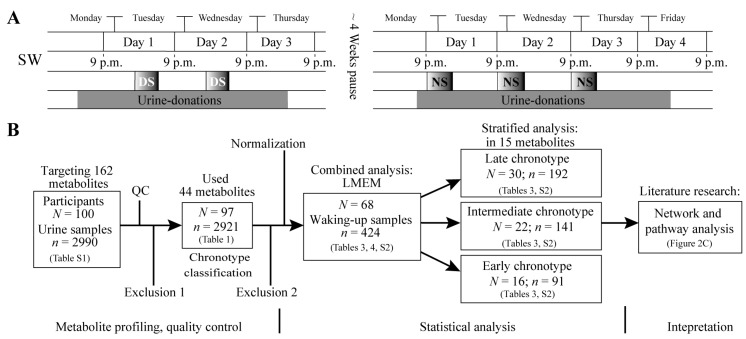
Study design and work flow for night shift and day shift comparison. Plot (**A**) shows an overview for nurses in SW (working both day and night shift) performing two shifts from Monday to Friday in day shift (DS) and night shift (NS) respectively with around a 4 week pause in between the study blocks. Boxes labelled DS indicate working hours in day shift, boxes labelled NS indicate working hours in night shift. Throughout the whole shift and observation period, urine samples were collected (grey boxes). Day 1 to day 4 lasts from 9 p.m. to 9 p.m. the next day and defines the time periods for comparison of day shift and night shift metabolic profiles; Plot (**B**) demonstrates an overview of the urine sample collection and exclusion, as well as the consecutive statistical and pathway analysis. Exclusion 1 = Exclude participants without information on sleep; Exclusion 2 = Exclude diabetics, vegetarians and participants with extreme sleep apnoea, as well as women working only day shift.

We recruited 100 study participants and used 97 women for a chronotype classification (Figure 1). These women had a mean age of 39.5 (25.0–60.0) and an average chronotype of 04:02 (01:17) (Table 1). Furthermore, we analysed 424 waking-up samples of 68 shift working (SW) nurses. The 68 SW nurses had mean age of 37.2 years (Table 1). We stratified the 68 SW nurses into early (*N* = 30), intermediate (*N* = 22) and late chronotype (*N* = 16) classes (Figure 1). The age of nurses in early and intermediate chronotype classes was comparable whereas nurses in the late chronotype class were younger (Table 1). We observed a negative correlation between age and chronotype values (Pearson correlation coefficient *r* = −0.45) (Figure S1). Moreover, early chronotypes smoked less and reported no respiratory diseases and a lower number of allergies when compared to late chronotypes. Respiratory diseases were most common (24%) among intermediate chronotypes (Table 1).

### 2.2. Correlation of 44 Metabolites Comparing Three Normalization Methods

Out of 162 quantified metabolites (Table S1), 44 metabolites passed our stringent quality control (see method). When comparing the measured creatinine concentration with the measured osmolality value in the 424 waking-up urine samples, we observed a moderate correlation (*r* = 0.72, *p*-value = 1.4 × 10^−4^). Furthermore, for the used 44 metabolites, we detected various correlation ranges among creatinine-, osmolality- and RBN normalized values (Table 2). The most significant correlation coefficients could be observed between RBN and creatinine-normalized values (*r* ranged from 0.48 to 0.99). The observed *r* ranged from 0.35 to 0.93 for the comparison of RBN with osmolality-normalized values and from −0.09 to 0.93 for the comparison between creatinine- and osmolality-normalized values (Table 2).

### 2.3. Metabolites Associated with Night Shift in the Combined Analysis in Three Normalization Methods

**Figure 2:**
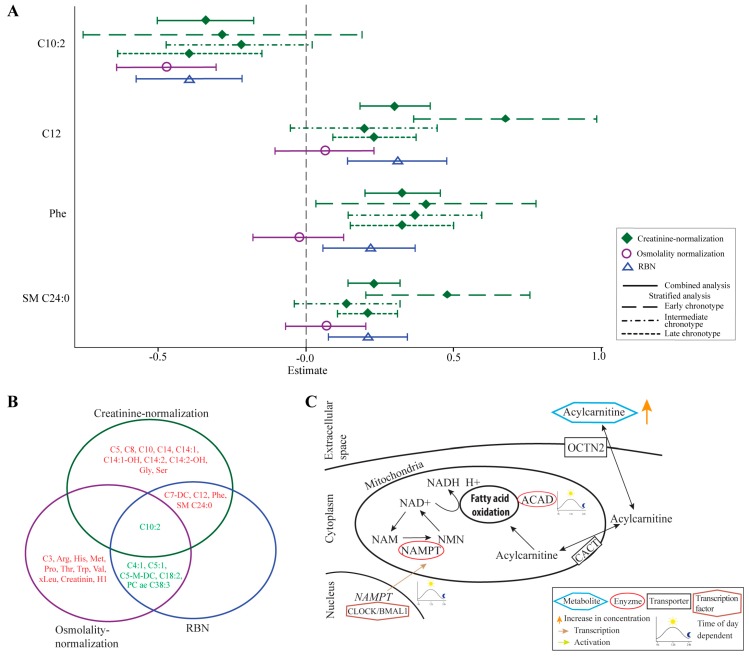
Results of four selected metabolites of three normalization methods of combined and chronotype-stratified analyses as well as pathways potentially affected by night shift work. Plot (**A**) shows the β-estimates and associated 95% confidence intervals of four metabolites based on creatinine-, osmolality- and regression based normalization of combined and stratified analysis; Plot (**B**) shows a Venn diagram of significantly altered metabolites when comparing DS with NS in the combined analysis of three normalization methods. Colours indicate the direction of the observed change (red = increase, green = decrease); Plot (**C**) depicts an overview for pathways potentially affected by night shift work. OCTN2, cell membrane carrier; CACT, Carnitine acylcarnitine translocase; NAD, Nicotinamide adenine dinucleotide (oxidised); NADH, Nicotinamide adenine dinucleotide (deoxidised); NAM, Nicotinamide; NMN, Nicotinamide mononucleotide; NAMPT, nicotinamide phosphoribosyl transferase; *NAMPT*, Gene encoding NAMPT.

Based on creatinine-normalized values, out of 44 analysed metabolites, urine concentrations of 15 metabolites were significantly altered between night and day shift, both in basic and full models (Table 3 and Table S2). The 15 metabolites consisted of 11 medium- and long-chain acylcarnitines (C5, C7-DC, C8, C10, C10:2, C12, C14, C14:1, C14:1-OH, C14:2, C14:2-OH), three amino acids (phenylalanine, glycine, serine) and one sphingomyelin (SM C24:0). With the exception of C10:2, we observed increased concentrations of the identified acylcarnitines in urine that was donated during night shift, when compared to urine from day shift (Figure 2A, Table 3 and Table S2).

Based on the osmolality-normalization, urine concentrations of 17 metabolites were significantly altered by night shift in both basic and full LMEM models (Table 4). The identified metabolites included six acylcarnitines, six amino acids, creatinine, PC ae C38:3 and hexose H1.

Using RBN, we found 10 significantly changed metabolites between night shift and day shift groups in both basic and full LMEM models. These 10 metabolites consisted of seven acylcarnitines, phenylalanine, PC ae C38:3 and SM C24:0 (Table 4).

We observed several consistent changes when comparing the three normalization methods. Acylcarnitine C10:2 was significantly decreased in night shift after applying any of the three normalization methods and the levels of nine metabolites were significantly altered in two out of three normalization methods (Figure 2B).

### 2.4. Metabolites Associated with Night Shift in the Chronotype—Stratified Analyses

We further investigated the influence of chronotypes on the concentration of 15 metabolites (identified with the creatinine normalization) in urine. We found that working night shifts significantly influenced 11, zero and four metabolites in nurses with early, intermediate and late chronotype, respectively (Table 3 and Table S2). In the early chronotype group, the 11 identified metabolites comprised 10 acylcarnitines (C5, C7-DC, C8, C10, C12, C14, C14:1, C14:1-OH, C14:2, C14:2-OH) and SM C24:0. In the late chronotype group, the levels of C10:2, C12, phenylalanine and SM C24:0 were changed when comparing night shift to day shift. Two metabolites, C12 and SM C24:0, were significantly altered both in early and late chronotype groups.

When comparing the results of combined and stratified analyses, 13 metabolites showed consistent changes in relation to night shift, whereas glycine and serine were only significant in the combined analysis (Table 3 and Table S2).

## 3. Discussion

Our main findings indicate that night shift work influences the metabolism of female nurses. The most changes concerning the metabolic profiles could be observed during night shift in nurses in the early chronotype class.

### 3.1. Identified Metabolites Largely Depend on the Applied Normalization Method

From a methodological point of view, our study is unique with respect to the application of three normalization methods to our multiple-time-point metabolomics data of nurses working in both night and day shifts.

Urine dilution can vary based upon water consumption and other physiological and pathophysiological factors, and, consequently, the concentrations of metabolites in urine also vary. Creatinine is a by-product of muscle metabolism. It is excreted from the body primarily through glomerular filtration. Creatinine is influenced by various factors, such as age, exercise but also physiological processes like the kidney tubule processing [19,22,23,24]. Creatinine -normalization is a commonly used approach, which makes comparison of results between studies feasible. To evaluate the total endogenous metabolic output in urine, osmolality can measured, which represents a direct measure thereof but can also be reduced by impaired kidney function [18,25,26].

Compared to creatinine, osmolality is usually not influenced by diurnal rhythms, diet, activity, age, stress or health state [18,27]. In our study, we observed a high correlation between measured creatinine and osmolality values, which is consistent with previous findings of *r* value of 0.75 [28]. However, regarding our used metabolite panel, the correlation coefficients between creatinine- and osmolality normalized values were low. We observed a low overlap of significant metabolites for creatinine and osmolality normalization, although we considered many influencing factors such as BMI and age in our statistical analysis.

We further present a new approach, RBN, which takes each metabolite’s excretion kinetic into account, allowing for a dilution correction per metabolite. Comparison of these normalization methods showed that they are appropriate for different research questions since they showed consistent results only for one metabolite, acylcarnitine C10:2. Creatinine-based and RBN methods seem particularly suitable for acyl carnitines, phosphatidylcholines and sphingomyelins whereas osmolality-based normalization seemed more suitable for amino acids. As to which normalization method to use depends on the study design and needs to be answered according to the research question. We focus on the creatinine normalization, as it has been used frequently and enables the comparison of our results with those of other studies.

### 3.2. Elevated Levels of Acylcarnitines May Result from Impaired Fatty Acid Oxidation

The increased urine levels of acylcarnitines during night shift could indicate an impairment of fatty acid oxidation. Acylcarnitines are imported and exported into/from the cell via the organic cation/carnitine transporter 2 (OCTN2) cell membrane carrier (Figure 2C) [29]. Due to the higher “social jet lag” and consequent sleep deprivation/restriction in nurses with an early chronotype, we hypothesize that these participants could be especially sensitive to night shift and show the most pronounced metabolic signs of sleep deprivation. Previous studies observed elevated blood levels of medium-chain acylcarnitines in healthy participants with acute sleep deprivation [9,10]. Due to an increased acylcarnitine concentration in blood, the renal OCTN2 carrier is saturated, yielding in a decreased renal reabsorption of acylcarnitines from urine [30]. We observed increased acylcarnitines concentration in urine during night shift, especially for nurses with early chronotype, which is in line with those previous reports. Furthermore, medium- and long-chain acylcarnitines produced in the kidney via fatty acid oxidation are directly secreted to the urine [31]. Inside mitochondria, the fatty acid oxidation is influenced by the NAD+ levels [8,12,15]. The nicotinamide phosphoribosyl transferase (NAMPT), a rate limiting enzyme of NAD+ synthesis, is regulated by the CLOCK/BMAL1 (Circadian Locomoter Output Cycles Kaput/Brain and Muscle Aryl hydrocarbon receptor nuclear translocator-Like 1) protein complex [32,33,34]. The transcription factor complex CLOCK/BMAL1 is a key modulator for the circadian rhythm [34]. The increased urine levels of acylcarnitines during night shift are likely to be due to a reduced circadian expression of NAMPT in shift working nurses (Figure 2C), leading to low NAD+ levels [32,33,35]. Moreover, acyl-CoA dehydrogenases (ACADs) catalyse the oxidation of long-chain fatty acids [36]. ACADs are subject to daily oscillations and prone to be influenced by shift work (Figure 2C) [36]. The metabolism of branched chain amino acids (BCAA) like leucine and isoleucine is catalysed via isovaleryl-CoA dehydrogenase, an enzyme from the ACAD family and might therefore be subject to perturbed circadian rhythms [37]. The observed medium chain acylcarnitine C14:2-OH is product of C14:2-OH-CoA which is metabolized by the bifunctional enzyme EHHADH (Enoyl-CoA Hydratase And 3-Hydroxyacyl CoA Dehydrogenase) [31]. The expression of EHHADH is highly dependent on the circadian clock gene *Bmal1*. Increased levels of C14:2-OH could be a sign of impaired activity of EHHADH via an reduced expression of Bmal1 in the kidney [31,38,39]. Additionally, a downregulation of *EHHADH* was associated with an impaired BCAA catabolism [40].

### 3.3. Strengths and Limitations

Our study has several advantages. First, our study is based on urine samples. As the collection of urine is non-invasive and does not required a medically trained expert. However, this matrix is significantly less standardized than blood. Therefore, we applied three normalization methods. Second, we used waking-up urine samples, which is a proxy of fasting samples. As the spontaneous urine samples were collected in the course of a real-life study, we could not obtain urine after the recommended 8-hour-fasting period. Third, the comparison of the metabolites profiles of nurses working in night shift and day shift was based on the identical group of individuals (68 nurses working in both shifts). Fourth, to account for differences in sex steroids and glucocorticoids, which reflect the stress or menstrual phase, we designed the study by including a 4 week pause between day shift and night shift.

Our study is limited by several factors. First, the number of participants used in our study was small, particularly in the stratified analysis. This led to a reduced statistical power. In order to address the problem of potential under- or over fitting, we applied two different sets of confounders. Metabolites reported in this study required to be significant in both settings (basic and full models). Second, the interpretation of our results is based on the proceedings and metabolite pathways from studies in cells or tissue models whereas the metabolites in our study were measured in urine that represents the last stage of metabolite degradation before its elimination. Moreover, metabolite concentrations may show tissue- and organ-specific regulation like kidney function or in muscle mass [23,24]. Third, we could not consider the potential influence of nutritional intake or of the duration of sleep to our multiple-time-point data. In our study, we tried to minimize this limitation by using waking-up samples. Fourth, we did not address potential diurnal changes which could influence the metabolite profiles. Reasons, therefore, were that many of our analysed samples were taken more than 12 h after the previous urine donation, which leads to an elimination of such diurnal effects. Fifth, our study was exclusively based on female participants.

### 3.4. Summary and Conclusions

Our results show an effect of night shift work on the metabolite values in urine. Out of 44 examined metabolites, 31 (about 70%) showed significantly altered concentrations by applying three normalization methods (creatinine-, osmolality- and regression-based normalization). We observed a low overlap of significant metabolites among the three methods. One metabolite was consistently identified with all three normalization methods and nine other metabolites (about 29%) were significantly altered in two out of three normalization methods. Individuals in the early chronotype class show the most significant metabolic changes. These were reflected by increased levels of acylcarnitines and altered concentrations of several amino acids in urine after night shifts. Independent studies, also including men, need to be conducted to confirm our finding that the individuals with intermediate or late chronotype classes show less affects in their metabolite profiles, when working in night shift.

## 4. Material and Methods

### 4.1. Study Design and Study Participants

For the current study, 100 female workers between 25 and 65 years of age were recruited at the clinical study site Bergmannsheil in Bochum, Germany. Exclusion criteria for study participation were (1) current pregnancy; (2) breastfeeding less than half a year ago; (3) past or present fertility medication; (4) prior cancer diagnosis.

After receiving a detailed explanation of the study protocol, each participant provided written informed consent. The study was conducted in accordance with the Declaration of Helsinki and the protocol was approved by the ethic review committee of the medical faculty at the Ruhr University Bochum, Germany (No. 3840-10).

The recruited study participants (*N* = 100) were assigned to shift working (SW) and non-shift working (non-SW) categories. The SW group (*N* = 75) consisted of nurses working both, day shift and night shift. A pause of four weeks between day and night shift assessments was scheduled to account for potential hormonal changes over time (such as the menstrual cycle) (Figure 1A). The non-SW group (*N* = 25) consisted of women working only day shift. During day shift, nurses worked for up to four consecutive days (Monday–Thursday) (for both SW and non-SW) and up to five consecutive days (Monday–Friday) during night shift. The core working time for nurses of the day shift was scheduled from 6 a.m.–2 p.m. (SW) and 8 a.m.–4 p.m. (non-SW), while the night shift lasted from 9 p.m.–6 a.m. (SW) (Figure 1A). The group of non-SW nurses was excluded from the further analysis of metabolite profiles as their sociodemographic characteristics and particularly their diverse disease states would not allow a reliable analysis and comparison of the metabolite profiles.

Throughout day shift and night shift, urine samples and information on diet, sleep and medication were collected.

### 4.2. Chronotype Classification

Participants’ chronotypes were calculated based on their sleep timing, as assessed by the Munich Chronotype Questionnaire for shift-workers (MCTQ^Shift^), an adaptation of the standard MCTQ [21,41]. Specifically, mid-sleep (the time point midway between falling asleep and waking up) on work-free days is a proxy for circadian phase that can be derived from entries to the MCTQ and chronotype then is defined as mid-sleep corrected for sleep debt accumulated over the past work week [21,41,42,43]. The MCTQ^Shift^ allows calculating mid-sleep times for different work shifts (e.g., early, late, night) and chronotype then is usually derived from the mid-sleep on work-free days after late shifts [21]. In our study, 97 women provided information about their habitual sleep times in different shifts. Twenty participants reported that they used an alarm clock on all work-free days in all work shifts. For these 20 participants, we used mid-sleep on workdays with late-shifts to calculate their chronotype. For three participants, we calculated chronotype based on their sleep-debt corrected mid-sleep on work-free days after early shifts. Data of all 97 participants were then grouped into early, intermediate and late chronotypes based on thirds (33.3%) of the sorted mid-sleep time points [21]. Nurses with chronotype <3:37 a.m. were defined as early chronotypes (*N* = 32), nurses with chronotype between 3:37 a.m.–4:25 a.m. were defined as intermediate chronotypes (*N* = 32) and nurses with chronotype >4:25 a.m. were defined as late chronotypes (*N* = 33).

### 4.3. Urine Samples

All spontaneous urine samples throughout observation period were collected in 100 mL SARSTEDT disposable plastic containers, stored at 9 °C for a maximum of 24 h before being aliquoted to 1.5 mL Eppendorf tubes and deep frozen at −80 °C. Out of the collected 3640 urine samples, 2990 were measured for metabolite profiles including 2921 samples of the 97 nurses with chronotype information. Due to potential influences on the metabolite profile and on the sleep quality, individuals with diabetes (*N* = 4), vegetarians (*N* = 2) and women with extreme sleep apnoea (*N* = 1) were excluded from the SW group, resulting in 68 participants with 2278 urine metabolite profiles [8,30,44]. Of those, 424 were logged by study participants as waking-up urine samples, indicating the first urine donation after waking up (Figure 1B). On average, waking-up urine samples were donated at 05:17 (SD = 01:17) a.m. during day shift and at 12:52 (SD = 03:04) p.m. for night shift.

### 4.4. Targeted Metabolite Profiling

Each urine sample was measured with the Absolute*IDQ*^TM^ p150 Kit (BIOCRATES Life Sciences AG, Innsbruck, Austria) using FIA-ESI-MS/MS (flow injection-electrospray ionisation-triple quadrupole mass spectrometry) [15,45]. In 10 µL urine, 162 metabolites were quantified (Table S1). We applied the same quality control (QC) criteria as in our previous study [15]. Overall, 2990 urine samples were measured in two batches. The QC was conducted separately for each metabolite and for each batch. In total, 44 metabolites passed the QC: free carnitine, 25 acylcarnitines (Cx:y), 13 proteinogenic amino acids, creatinine, hexoses (sum of hexoses), two phosphatidylcholine acyl-alkyl (PC ae) and one sphingolipid (SM C24:0) (Table S1). The abbreviations Cx:y depicts x number of carbons and y double bonds of all chains, respectively.

### 4.5. Osmolality Measurement

Measurements were performed using a Gonotec Osmomat 030 (Berlin, Germany). Freezing-point depression was used to determine osmolality [osm/kg] in waking-up urine samples.

### 4.6. Normalization Approaches

Urine samples where normalized to the respective creatinine and osmolality values, respectively [46,47]. Furthermore, a novel approach, regression based normalization (RBN), was applied [48]. In RBN, the dependency of urinary metabolite on the dilution variation is estimated from the data which allows for nonlinear functional relations. Nonlinear relations can occur in dynamically influenced urinary data.

### 4.7. Statistical Analysis

Normalized metabolite values were log-transformed and standardized (mean = 0 and standard deviation = 1). We used LMEM to compare the metabolic profiles between day shift (used as reference in the current study) and night shift of the SW group. A combined analysis, based on metabolite values of 68 participants, as well as a stratified analysis based on the respective chronotype class (early, intermediate and late) was conducted. For each metabolite, we calculated basic and full LMEMs. The basic model was adjusted for chronotype value and batch effect. To account for the sociodemographic differences between the study groups, the full model was additionally adjusted for BMI, age, smoking status, thyroid disease status, total years of shift work, respective day of shift block and time since last urination [12,49,50].

To account for multiple testing of the 44 used metabolites, false discovery rate (FDR, Benjamini Hochberg) was used as significance cut-off.

Statistical analyses were performed with SAS 9.4 (SAS Institute, Cary, NC, USA).

## Figures and Tables

**Table 1:** Characteristics of participants. Characteristics of all participants, shift working group (SW) in combined and stratified analyses are shown. Means with standard deviations (SD) or number of phenotypes with percentages are shown for each group. BMI (body mass index). * Chronotype is defined as mid-sleep corrected for sleep debt accumulated over the past work week.

Clinical Parameters	All Participants	Shift Working Participants (Combined Analysis)	Stratified Analysis		
			Early Chronotype	Intermediate Chronotype	Late Chronotype
N	97	68	16	22	30
Chronotype (SD) *, a.m.	04:02 (01:17)	04:21 (01:14)	02:50 (00:43)	03:59 (00:14)	05:26 (00:48)
Mean age (range), years	39.5 (25.0–60.0)	37.2 (25.0–57.0)	41.3 (25.0–50.0)	40.5 (25.0–57.0)	32.5 (25.0–56.0)
BMI, kg/m^2^	26.2 (5.2)	26.2 (5.0)	26.6 (4.5)	26.7 (5.5)	25.7 (5.0)
Regular smoker (%)	27 (27.8)	26 (38.2)	3 (18.8)	10 (45.5)	13 (43.3)
Thyroid disease (%)	20 (20.1)	12 (17.6)	4 (25.0)	2 (9.1)	6 (19.4)
Hypertension (%)	16 (16.5)	10 (14.7)	2 (12.5)	3 (13.6)	5 (16.7)
Respiratory disease (%)	14 (14.4)	9 (13.4)	0 (0.0)	5 (23.8)	4 (13.3)
Cases of Allergy (%)	53 (54.6)	37 (54.4)	6 (37.5)	11 (50.0)	20 (66.7)
Kidney disease (%)	2 (2.1)	0 (0.0)	0 (0.0)	0 (0.0)	0 (0.0)

**Table 2:** Correlation of 44 metabolites concentrations after creatinine-, osmolality- and regression-based normalization. Pearson correlation coefficients of three pairwise comparisons are shown. Non-significant correlations are indicated in bold (Bonferroni cut off; *p*-values < 1.14 × 10^−3^). In summary, absolute correlation coefficients > 0.15 reflect a statistical significance.

Metabolite	Creatinine Normalization vs. Osmolality Normalization	Osmolality Normalization vs. RBN	RBN vs. Creatinine Normalization
C0	0.76	0.84	0.97
C2	0.84	0.85	0.98
C3	0.68	0.76	0.96
C4:1	0.93	0.93	0.99
C5	0.73	0.89	0.93
C5-M-DC	0.75	0.83	0.96
C5:1	0.62	0.79	0.93
C5:1-DC	0.68	0.76	0.95
C6:1	**0.13**	0.55	0.71
C7-DC	0.21	0.56	0.79
C8	−0.01	0.49	0.61
C8:1	0.70	0.72	0.96
C9	0.65	0.77	0.93
C10	**−0.08**	0.49	0.57
C10:1	0.18	0.49	0.79
C10:2	0.74	0.82	0.96
C12	**0.09**	0.63	0.64
C14	0.16	0.66	0.56
C14:1	0.23	0.70	0.70
C14:1-OH	0.16	0.70	0.61
C14:2	**0.12**	0.64	0.62
C14:2-OH	**0.05**	0.61	0.57
C16	0.47	0.78	0.77
C16-OH	0.68	0.90	0.86
C16:2	0.19	0.75	0.48
C18:2	0.32	0.76	0.49
Arg	0.33	0.44	0.80
Gln	0.44	0.50	0.86
Gly	0.64	0.60	0.92
His	0.58	0.51	0.90
Met	**−0.09**	0.35	0.61
Phe	0.42	0.52	0.87
Pro	0.22	0.42	0.71
Ser	0.44	0.55	0.85
Thr	0.50	0.53	0.89
Trp	0.21	0.41	0.81
Tyr	0.48	0.49	0.88
Val	0.28	0.36	0.75
Leu/Isoleu	0.45	0.36	0.76
Creatinine	-	0.20	-
PC ae C38:3	0.44	0.83	0.61
PC ae C38:6	**0.11**	0.55	0.66
SM C24:0	0.44	0.80	0.72
H1	0.50	0.59	0.88

**Table 3:** Results of 15 metabolites significantly altered by night shift work in combined and chronotype-stratified analysis based on creatinine-normalized values in the fully adjusted model. For each metabolite, the β-estimate, the 95% confidence interval (CI) and false discovery rate (FDR) of the full linear mixed effect model (LMEM) for the comparison of day shift (reference) and night shift are shown. The full linear model was adjusted for chronotype, batch effect, smoking status, age, BMI, thyroid disease status, total years of shift work, day of shift and time since last urination. Significant *p*-values (FDR < 0.05) in both basic and full LMEM model are indicated in bold. *N* = Number of nurses; *n* = Number of urine samples.

	Combined Analysis *N* = 68; *n* = 424		Early Chronotype *N* = 16; *n* = 91		Intermediate Chronotype *N* = 22; *n* = 141		Late Chronotype *N* = 30; *n* = 192	
Metabolites	β–Estimate (95% CI)	FDR *p*-value	β–Estimate (95% CI)	FDR *p*-value	β-Estimate (95% CI)	FDR *p*-Value	β-Estimate (95% CI)	FDR *p*-Value
C5	0.09 (0.03, 0.15)	**1.8 × 10^−2^**	0.24 (0.10, 0.38)	**6.3 × 10^−3^**	−0.02 (−0.12, 0.09)	0.87	0.10 (0.01, 0.19)	0.12
C7-DC	0.23 (0.12, 0.35)	**7.1 × 10^−4^**	0.53 (0.25, 0.81)	**4.3 × 10^−3^**	0.14 (−0.08, 0.35)	0.47	0.15 (−0.01, 0.31)	0.23
C8	0.15 (0.05, 0.26)	**1.7 × 10^−2^**	0.51 (0.23, 0.79)	**4.3 × 10^−3^**	0.00 (−0.21, 0.20)	0.99	0.16 (0.02, 0.29)	0.12
C10	0.18 (0.07, 0.28)	**4.7 × 10^−3^**	0.57 (0.26, 0.88)	**4.3 × 10^−3^**	0.05 (−0.15, 0.25)	0.83	0.14 (0.01, 0.27)	0.12
C10:2	−0.34 (−0.50, −0.18)	**5.1 × 10^-4^**	−0.22 (−0.69, 0.24)	0.46	−0.22 (−0.47, 0.03)	0.38	−0.39 (−0.64, −0.14)	**2.3 × 10^−2^**
C12	0.30 (0.18, 0.42)	**1.4 × 10^−5^**	0.68 (0.37, 0.99)	**2.0 × 10^−3^**	0.19 (−0.06, 0.44)	0.41	0.23 (0.09, 0.37)	**2.3 × 10^−2^**
C14	0.16 (0.04, 0.27)	**2.1 × 10^−2^**	0.53 (0.19, 0.86)	**8.7 × 10^−3^**	0.09 (−0.13, 0.32)	0.66	0.04 (−0.10, 0.19)	0.78
C14:1	0.18 (0.07, 0.28)	**4.7 × 10^−3^**	0.52 (0.23, 0.82)	**4.6 × 10^−3^**	0.14 (−0.06, 0.35)	0.45	0.04 (−0.09, 0.17)	0.78
C14:1-OH	0.21 (0.09, 0.33)	**4.7 × 10^−3^**	0.58 (0.23, 0.93)	**6.6 × 10^−3^**	0.20 (−0.04, 0.43)	0.41	0.05 (−0.10, 0.20)	0.78
C14:2	0.18 (0.07, 0.29)	**6.6 × 10^−3^**	0.50 (0.17, 0.82)	**1.1 × 10^−2^**	0.13 (−0.07, 0.34)	0.47	0.08 (−0.06, 0.22)	0.52
C14:2-OH	0.16 (0.05, 0.28)	**1.8 × 10^−2^**	0.56 (0.22, 0.91)	**7.6 × 10^−3^**	0.09 (−0.13, 0.30)	0.66	0.04 (−0.10, 0.18)	0.81
Gly	0.16 (0.05, 0.28)	**1.8 × 10^−2^**	−0.09 (−0.35, 0.17)	0.65	0.27 (0.07, 0.46)	0.10	0.21 (0.04, 0.39)	0.12
Phe	0.33 (0.20, 0.45)	**1.4 × 10^−5^**	0.40 (0.03, 0.78)	8.4 × 10^−2^	0.37 (0.14, 0.60)	6.9 × 10^−2^	0.32 (0.15, 0.50)	**8.0 × 10^−3^**
Ser	0.15 (0.03, 0.27)	**3.8 × 10^−2^**	−0.06 (−0.35, 0.23)	0.77	0.22 (0.02, 0.43)	0.29	0.17 (−0.02, 0.36)	0.25
SM C24:0	0.23 (0.14, 0.32)	**1.4 × 10^−5^**	0.48 (0.20, 0.76)	**5.3 × 10^−3^**	0.14 (−0.04, 0.32)	0.41	0.21 (0.11, 0.31)	**4.1 × 10^−3^**

**Table 4:** Metabolites associated with night shift (osmolality- and regression-based normalized values). The table shows β-estimates and false discovery rate (FDR) values for 21 metabolites which are significantly associated with night shift work. The calculations were based on osmolality and regression-based normalizations. The basic LMEM was adjusted for chronotype value and batch effect. The full model was adjusted for chronotype value, batch effect, BMI, age, smoking status, thyroid disease status, total years of shift work, day of shift and time since last urination. Significant FDR values are indicated in bold.

	Osmolality-Normalization				Regression Based Normalization			
Metabolite	Basic Model		Full model		Basic Model		Full model	
	β-Estimate (95% CI)	FDR	β-Estimate (95% CI)	FDR	β-Estimate (95% CI)	FDR	β-Estimate (95% CI)	FDR
C3	−0.23 (−0.39, −0.07)	**2.0 × 10^−2^**	−0.25 (−0.42, −0.07)	**1.9 × 10^−2^**	−0.14 (−0.29, 0.02)	0.17	−0.15 (−0.32, 0.01)	0.17
C4:1	−0.10 (−0.16, −0.04)	**3.7 × 10^−3^**	−0.10 (−0.16, −0.05)	**4.2 × 10^−3^**	−0.07 (−0.12, −0.02)	**2.1 × 10^−2^**	−0.07 (−0.12, −0.02)	**4.5 × 10^−2^**
C5-M-DC	−0.26 (−0.37, −0.14)	**2.5 × 10^−4^**	−0.29 (−0.41, −0.16)	**1.1 × 10^−4^**	−0.22 (−0.32, −0.11)	**5.9 × 10^−4^**	−0.24 (−0.35, −0.13)	**4.6 × 10^−4^**
C5:1	−0.27 (−0.42, −0.12)	**3.5 × 10^−3^**	−0.32 (−0.48, −0.16)	**9.2 × 10^−4^**	−0.19 (−0.33, −0.06)	**2.1 × 10^−2^**	−0.25 (−0.39, −0.11)	**4.7 × 10^−3^**
C7-DC	−0.05 (−0.21, 0.11)	0.62	−0.04 (−0.21, 0.14)	0.72	0.26 (0.10, 0.42)	**1.1 × 10^−2^**	0.29 (0.12, 0.46)	**6.9 × 10^−3^**
C10:2	−0.49 (−0.65, −0.33)	**1.6 × 10^−7^**	−0.47 (−0.64, −0.30)	**4.3 × 10^−6^**	−0.42 (−0.58, −0.25)	**2.0 × 10^−5^**	−0.39 (−0.57, −0.22)	**4.1 × 10^−4^**
C12	0.03 (−0.12, 0.19)	0.72	0.06 (−0.11, 0.23)	**0.51**	0.25 (0.09, 0.41)	**1.2 × 10^−2^**	0.31 (0.14, 0.48)	**3.5 × 10^−3^**
C18:2	−0.19 (−0.34, −0.04)	**3.5 × 10^−2^**	−0.23 (−0.39, −0.07)	**1.8 × 10^−2^**	−0.25 (−0.40, −0.09)	**1.1 × 10^−2^**	−0.32 (−0.48, −0.15)	**1.7 × 10^−3^**
Arg	−0.29 (−0.44, −0.15)	**8.8 × 10^−4^**	−0.31 (−0.46, −0.16)	**9.2 × 10^−4^**	−0.20 (−0.36, −0.04)	6.2 × 10^−2^	−0.26 (−0.43, −0.08)	**2.1 × 10^−2^**
His	−0.15 (−0.28, −0.03)	**3.8 × 10^−2^**	−0.16 (−0.29, −0.03)	**4.3 × 10^−2^**	−0.03 (−0.15, 0.08)	0.70	−0.05 (−0.17, 0.08)	0.56
Met	−0.23 (−0.38, −0.07)	**1.7 × 10^−2^**	−0.24 (−0.40, −0.08)	**1.4 × 10^−2^**	−0.13 (−0.30, 0.03)	0.21	−0.16 (−0.33, 0.02)	0.18
Phe	0.00 (−0.15, 0.14)	0.98	−0.02 (−0.18, 0.13)	0.76	0.24 (0.09, 0.39)	**1.1 × 10^−2^**	0.21 (0.06, 0.37)	**3.6 × 10^−2^**
Pro	−0.24 (−0.38, −0.09)	**9.9 × 10^−3^**	−0.25 (−0.41, −0.10)	**7.0 × 10^−3^**	−0.05 (−0.19, 0.09)	0.61	−0.09 (−0.24, 0.06)	0.36
Thr	−0.20 (−0.36, −0.05)	**3.2 × 10^−2^**	−0.22 (−0.38, −0.06)	**2.7 × 10^−2^**	−0.09 (−0.23, 0.05)	0.42	−0.12 (−0.27, 0.04)	0.28
Trp	−0.19 (−0.32, −0.06)	**2.0 × 10^−2^**	−0.21 (−0.36, −0.07)	**1.3 × 10^−2^**	−0.01 (−0.12, 0.11)	0.90	−0.05 (−0.17, 0.07)	0.56
Val	−0.18 (−0.33, −0.03)	**4.1 × 10^−2^**	−0.20 (−0.36, −0.04)	**4.1 × 10^−2^**	−0.07 (−0.22, 0.09)	0.56	−0.11 (−0.28, 0.05)	0.31
Leu/Isoleu	−0.25 (−0.40, −0.10)	**7.6 × 10^−3^**	−0.27 (−0.43, −0.11)	**5.1 × 10^−3^**	−0.09 (−0.24, 0.07)	0.49	−0.13 (−0.30, 0.04)	0.28
Creatinine	−0.18 (−0.33, −0.04)	**3.4 × 10^−2^**	−0.19 (−0.34, −0.04)	**3.5 × 10^−2^**	−0.15 (−0.30, 0.00)	0.12	−0.18 (−0.34, −0.02)	**9.3 × 10^−2^**
PC ae C38:3	−0.35 (−0.51, −0.18)	**6.8 × 10^−4^**	−0.34 (−0.51, −0.16)	**2.1 × 10^−3^**	−0.57 (−0.74, −0.40)	**4.5 × 10^−9^**	−0.55 (−0.74, −0.37)	**2.0 × 10^−7^**
SM C24:0	0.05 (−0.07, 0.18)	0.45	0.07 (−0.07, 0.20)	0.39	0.19 (0.06, 0.31)	**1.9 × 10^−2^**	0.21 (0.08, 0.34)	**1.2 × 10^−2^**
H1	−0.26 (−0.40, −0.11)	**3.7 × 10^−3^**	−0.25 (−0.40, −0.10)	**7.0 × 10^−3^**	−0.20 (−0.36, −0.04)	0.62	−0.17 (−0.34, 0.01)	0.15

## Supplementary Materials

The following are available online at http://www.mdpi.com/2218-1989/8/3/45/s1, Figure S1. Correlation plot for age and chronotype value, Table S1. List of 162 quantified metabolites, Table S2. Results of 15 metabolites significantly altered by night shift work in combined and chronotype-stratified analysis based on creatinine-normalized values in the basic model.
