# Supplementary material for: Night Shift Work Affects Urine Metabolite Profiles of Nurses with Early Chronotype
Source: Metabolites. 2018 Aug 21;8(3):45. doi: 10.3390/metabo8030045 (PMC6161245; doi:10.3390/metabo8030045)
Supplement: Supplementary file 1 [file metabolites-08-00045-s001.pdf]

Supplementary material

Figure S1. Correlation plot for age and chronotype value

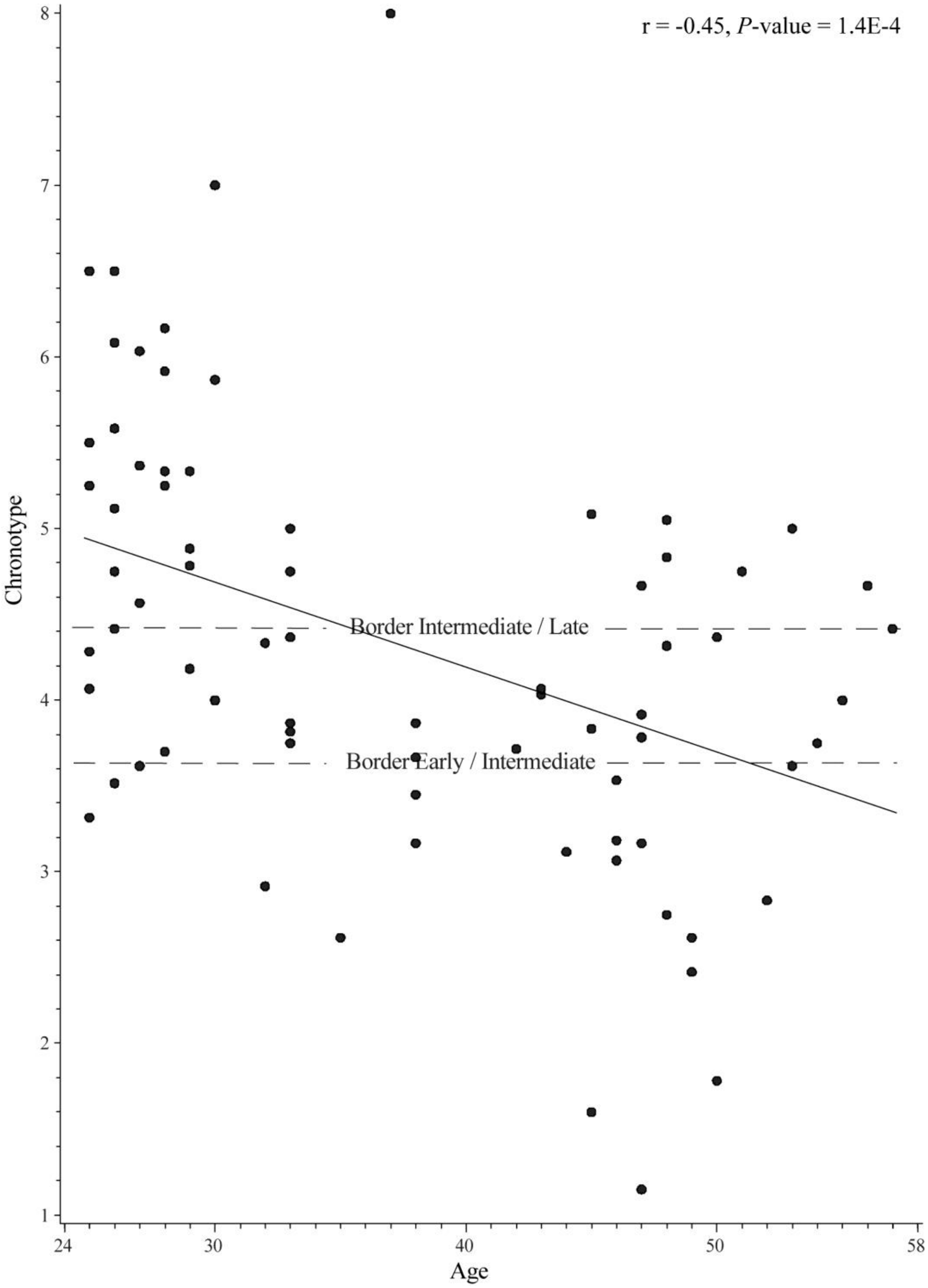

Pearson correlation plot between age and chronotype for nurses doing both day and night shifts (solid black dots) (N=68). Dashed lines indicate the separation into the strata early and intermediate (border early / intermediate) and intermediate and late (border intermediate / late) chronotypes. The continuous line represents the regression line (equation: chronotype value = 6.3522243889 – 0.053634583*age).

Table S1. List of 162 quantified metabolites

The first column shows the name of the metabolite. Application indicates if the respective metabolite passed the quality controls and whether it was included or excluded from further analysis. The third to eighth column illustrate the percentage of concentrations of all samples in both batches below the limit of detection (LOD), their coefficient of variance (CV) and mean concentrations with standard deviation (SD).

		**Measurement batch 1**			**Measurement batch 2**		
Metabolites	Application	Measurements below LOD (%)	CV (%)	Mean concentration (SD) (μM)	Measurements below LOD (%)	CV (%)	Mean concentration (SD) (μM)
**Acylcarnitines**							
C0	Used	2.9	14.7	45.48 (52.244)	2.9	7.4	55.730 (56.108)
C2	Used	0.1	16.3	16.509 (22.147)	0.0	7.6	17.939 (20.426)
C3	Used	1.3	15.4	0.695 (0.725)	1.5	7.8	0.932 (0.826)
C3:OH	Excluded	2.9	25.0	0.66 (0.473)	53.2	18.0	0.059 (0.058)
C3:1	Excluded	62.0	28.9	0.05 (0.044)	28.8	16.8	0.021 (0.011)
C4	Excluded	59.9	454.0	0.085 (0.522)	0.0	6.8	5.108 (4.025)
(C3-DC)C4-OH	Excluded	1.7	52.5	0.186 (0.173)	2.4	9.5	0.946 (0.646)
C4:1	Used	0.7	24.8	5.136 (4.268)	2.4	9.1	0.164 (0.141)
C5	Used	0.6	16.0	0.195 (0.147)	2.0	7.6	0.237 (0.203)
C5-DC (C6-OH)	Excluded	1.8	54.7	0.596 (0.738)	1.5	8.5	2.121 (1.587)
C5-M-DC	Used	0.3	23.4	1.825 (1.691)	1.0	10.9	0.624 (0.494)
C5-OH (C3-DC-M)	Excluded	0.3	48.0	0.428 (0.318)	2.4	10.4	1.100 (3.589)
C5:1	Used	0.7	22.7	0.579 (1.647)	2.4	8.5	1.048 (0.701)
C5:1-DC	Used	0.5	19.7	0.754 (0.546)	2.4	9.9	0.310 (0.219)
C6 (C4:1-DC)	Excluded	0.8	27.9	0.246 (0.2)	2.4	9.2	0.613 (0.582)
C6:1	Used	11.8	15.4	0.141 (0.104)	5.9	11.4	0.139 (0.116)
C7-DC	Used	1.6	15.0	0.253 (0.229)	2.4	10.3	0.235 (0.226)
C8	Used	15.3	13.9	0.456 (0.318)	10.2	11.4	0.453 (0.415)
C8:1	Used	0.1	17.3	3.561 (4.197)	0.5	10.8	3.013 (3.662)
C9	Used	0.1	15.1	2.913 (3.016)	0.0	6.0	2.772 (2.642)
C10	Used	16.0	13.7	0.235 (0.149)	7.8	6.8	0.284 (0.223)
C10:1	Used	0.3	16.4	0.812 (0.661)	2.0	6.4	0.834 (0.627)
C10:2	Used	0.1	18.7	1.443 (3.603)	1.0	5.9	1.052 (1.449)
C12	Used	10.6	13.0	0.259 (0.178)	5.4	6.8	0.296 (0.215)
C12:DC	Excluded	99.8	8.6	0.173 (0.074)	99.5	6.3	0.127 (0.038)
C12:1	Excluded	73.7	13.4	0.259 (0.164)	51.2	7.3	0.271 (0.168)
C14	Used	49.2	14.5	0.025 (0.018)	35.1	8.1	0.038 (0.049)
C14:1	Used	24.4	17.3	0.015 (0.013)	14.6	14.1	0.025 (0.029)
C14:1-OH	Used	39.3	15.2	0.016 (0.012)	20.5	10.7	0.020 (0.022)
C14:2	Used	22.5	16.5	0.014 (0.012)	7.3	12.7	0.022 (0.026)
C14:2-OH	Used	31.9	16.9	0.013 (0.009)	22.0	12.5	0.018 (0.019)
C16	Used	36.2	21.6	0.04 (0.21)	29.3	9.7	0.077 (0.469)
C16:OH	Used	47.5	22.5	0.031 (0.179)	14.6	10.2	0.076 (0.401)
C16:1	Excluded	99.7	11.8	0.041 (0.014)	99.5	10.0	0.063 (0.025)
C16:1-OH	Excluded	69.3	19.6	0.007 (0.004)	51.7	17.1	0.009 (0.010)
C16:2	Used	37.9	22.1	0.01 (0.007)	22.4	10.7	0.013 (0.009)
C16:2-OH	Excluded	90.9	22.7	0.01 (0.003)	94.1	13.1	0.014 (0.006)
C18	Excluded	73.3	22.2	0.006 (0.004)	68.8	14.6	0.009 (0.008)
C18:1	Excluded	91.7	17.8	0.007 (0.003)	91.2	19.1	0.008 (0.003)
C18:1-OH	Excluded	98.0	18.2	0.009 (0.002)	99.5	11.4	0.012 (0.003)
C18:2	Used	44.7	22.5	0.005 (0.003)	48.3	9.2	0.006 (0.004)
**Amino acids**							
Arg	Used	15.5	21.3	15.165 (12.555)	4.9	9.6	17.480 (14.599)
Gln	Used	0.1	18.6	251.184 (227.606)	0.0	5.9	258.361 (173.577)
Gly	Used	0.1	19.2	771.854 (748.743)	0.0	8.0	897.190 (1185.140)
His	Used	0.1	18.4	367.353 (397.542)	0	7.8	315.812 (228.959)
Met	Used	2.8	20.8	9.393 (6.659)	3.4	12.7	9.373 (6.106)
Phe	Used	0.1	16.1	18.527 (16.473)	0	5.8	19.804 (12.093)
Pro	Used	0.5	17.9	16.972 (13.35)	2.4	9.4	15.162 (12.098)
Ser	Used	0.1	19.7	180.859 (166.863)	1.0	17.9	179.772 (123.364)
Thr	Used	0.1	20.9	78.284 (81.509)	1.5	18.6	67.670 (49.736)
Trp	Used	8.2	16.5	44.342 (38.189)	3.9	6.9	52.080 (33.292)
Tyr	Used	0.1	17.8	64.432 (68.57)	2.0	7.6	66.398 (50.392)
Val	Used	0.2	19.1	27.398 (22.034)	2.4	6.9	26.581 (17.557)
Leu/Isoleu	Used	0.1	19.7	43.022 (39.313)	2.4	7.1	43.256 (33.167)
Creatinine	Used	0.1	14.9	7000.62 (39818.44)	0	6.0	6644.470 (5104.090)
**Phosphatidylcholines**							
PC aa C24:0	Excluded	99.8	32.5	0.016 (0.008)	99.0	14.2	0.019 (0.036)
PC aa C26:0	Excluded	100.0	3.5	0.397 (0.016)	100.0	3.2	0.398 (0.017)
PC aa C28:1	Excluded	89.5	26.1	0.058 (0.03)	22.0	13.1	0.062 (0.038)
PC aa C30:0	Excluded	99.9	9.9	0.051 (0.009)	100.0	6.4	0.051 (0.010)

PC aa C30:2	Excluded	44.9	63.7	0.002 (0.003)	25.9	57.6	0.002 (0.002)
PC aa C32:0	Excluded	80.5	26.1	0.016 (0.022)	70.7	9.7	0.013 (0.017)
PC aa C32:1	Excluded	70.4	43.1	0.009 (0.016)	74.1	32.6	0.007 (0.015)
PC aa C32:2	Excluded	76.8	61.3	0.002 (0.003)	77.1	47.8	0.002 (0.004)
PC aa C32:3	Excluded	98.2	51.3	0.002 (0.001)	74.1	53.9	0.002 (0.001)
PC aa C34:1	Excluded	23.2	40.8	0.08 (0.136)	11.7	16.7	0.078 (0.203)
PC aa C34:2	Excluded	79.2	60.4	0.062 (0.23)	17.1	30.3	0.073 (0.419)
PC aa C34:3	Excluded	98.5	39.2	0.006 (0.01)	96.1	28.8	0.004 (0.012)
PC aa C34:4	Excluded	91.8	30.1	0.007 (0.005)	69.3	25.7	0.006 (0.009)
PC aa C36:0	Excluded	100.0	15.0	0.053 (0.006)	100.0	8.8	0.045 (0.005)
PC aa C36:1	Excluded	21.7	35.4	0.035 (0.042)	14.6	21.2	0.037 (0.062)
PC aa C36:2	Excluded	32.3	62.2	0.045 (0.119)	26.8	26.8	0.055 (0.248)
PC aa C36:3	Excluded	32.4	50.8	0.026 (0.083)	15.1	20.9	0.030 (0.135)
PC aa C36:4	Excluded	48.5	73.4	0.024 (0.094)	26.3	26.8	0.032 (0.192)
PC aa C36:5	Excluded	95.7	45.7	0.004 (0.008)	93.7	30.8	0.004 (0.014)
PC aa C36:6	Excluded	32.4	84.3	0.002 (0.002)	26.3	43.1	0.003 (0.003)
PC aa C38:0	Excluded	99.7	23.3	0.014 (0.004)	98.5	15.2	0.012 (0.005)
PC aa C38:1	Excluded	86.6	65.6	0.004 (0.004)	83.4	68.6	0.005 (0.004)
PC aa C38:3	Excluded	64.5	55.4	0.009 (0.024)	37.6	41.2	0.012 (0.045)
PC aa C38:4	Excluded	43.4	66.1	0.017 (0.049)	24.9	59	0.022 (0.118)
PC aa C38:5	Excluded	68.3	59.0	0.007 (0.019)	62.9	58.7	0.010 (0.045)
PC aa C38:6	Excluded	45.5	51.9	0.011 (0.025)	18.5	36.3	0.013 (0.049)
PC aa C40:1	Excluded	100.0	21.0	0.163 (0.033)	100.0	7.9	0.132 (0.014)
PC aa C40:2	Excluded	99.9	26.2	0.01 (0.003)	98.5	26.4	0.009 (0.003)
PC aa C40:3	Excluded	94.6	45.9	0.002 (0.002)	74.6	40	0.003 (0.002)
PC aa C40:4	Excluded	99.6	30.3	0.005 (0.002)	98.5	27.1	0.006 (0.005)
PC aa C40:5	Excluded	92.8	315.1	0 (0.004)	92.2	387.3	0.001 (0.011)
PC aa C40:6	Excluded	99.9	9.9	0.074 (0.01)	99.0	4.8	0.077 (0.019)
PC aa C42:0	Excluded	100.0	15.5	0.025 (0.006)	100.0	13.2	0.025 (0.008)
PC aa C42:1	Excluded	100.0	38.8	0.008 (0.003)	100.0	36.1	0.007 (0.002)
PC aa C42:2	Excluded	100.0	11.8	0.037 (0.005)	100.0	10.8	0.033 (0.004)
PC aa C42:4	Excluded	95.4	40.5	0.004 (0.002)	100.0	54.6	0.004 (0.002)
PC aa C42:5	Excluded	98.4	38.4	0.009 (0.004)	78.0	50.1	0.006 (0.003)
PC aa C42:6	Excluded	100.0	27.4	0.099 (0.028)	100.0	14.4	0.098 (0.020)
PC ae C30:0	Excluded	100.0	21.9	0.053 (0.011)	100.0	9.6	0.042 (0.006)
PC ae C30:1	Excluded	98.6	46.5	0.006 (0.003)	36.6	137.1	0.002 (0.003)
PC ae C30:2	Excluded	99.9	13.3	0.025 (0.006)	100.0	5.6	0.109 (0.009)
PC ae C32:1	Excluded	63.9	44.0	0.005 (0.006)	63.4	34.5	0.004 (0.007)
PC ae C32:2	Excluded	100.0	19.7	0.012 (0.004)	100.0	21.6	0.008 (0.003)
PC ae C34:0	Excluded	97.8	28.8	0.008 (0.004)	93.2	24.9	0.006 (0.004)
PC ae C34:1	Excluded	68.1	23.3	0.015 (0.019)	61.0	21.5	0.013 (0.018)
PC ae C34:2	Excluded	51.6	42.6	0.006 (0.012)	19.5	31.6	0.006 (0.012)
PC ae C34:3	Excluded	92.2	41.3	0.006 (0.005)	63.9	33.4	0.005 (0.008)
PC ae C36:0	Excluded	100.0	14.6	0.046 (0.019)	100.0	10.8	0.038 (0.015)
PC ae C36:1	Excluded	99.8	26.7	0.034 (0.013)	98.0	18.2	0.023 (0.014)
PC ae C36:2	Excluded	95.8	19.5	0.017 (0.013)	95.1	18.6	0.015 (0.012)
PC ae C36:3	Excluded	93.8	48.3	0.005 (0.006)	65.9	29	0.004 (0.007)
PC ae C36:4	Excluded	64.9	29.3	0.011 (0.009)	73.7	31.1	0.009 (0.014)
PC ae C36:5	Excluded	98.9	36.4	0.006 (0.006)	93.2	20.3	0.007 (0.015)
PC ae C38:0	Excluded	100.0	9.6	0.051 (0.007)	100.0	5.9	0.045 (0.008)
PC ae C38:1	Excluded	91.0	72.6	0.002 (0.003)	93.2	93.7	0.002 (0.004)
PC ae C38:2	Excluded	74.8	56.2	0.004 (0.004)	75.1	50	0.004 (0.005)
PC ae C38:3	Used	48.5	23.5	0.017 (0.009)	32.7	18.0	0.021 (0.016)
PC ae C38:4	Excluded	48.5	62.7	0.004 (0.007)	42.4	41.6	0.005 (0.012)
PC ae C38:5	Excluded	100.0	15.2	0.103 (0.031)	100.0	14.6	0.106 (0.056)
PC ae C38:6	Used	0.8	19.7	0.052 (0.035)	1.5	12.9	0.053 (0.035)
PC ae C40:1	Excluded	99.4	29.7	0.007 (0.003)	98.0	35.8	0.007 (0.003)
PC ae C40:2	Excluded	58.4	34.0	0.005 (0.004)	52.7	27.1	0.005 (0.004)
PC ae C40:3	Excluded	87.4	75.7	0.002 (0.003)	48.8	92.7	0.003 (0.006)
PC ae C40:4	Excluded	99.9	12.6	0.029 (0.005)	99.5	12.2	0.030 (0.009)
PC ae C40:5	Excluded	88.2	66.6	0.003 (0.003)	91.7	41.8	0.003 (0.007)
PC ae C40:6	Excluded	99.4	40.1	0.005 (0.003)	98.0	38.2	0.005 (0.004)
PC ae C42:0	Excluded	100.0	6.0	0.478 (0.03)	100.0	3.5	0.418 (0.022)
PC ae C42:1	Excluded	100.0	12.1	0.054 (0.007)	100.0	7.6	0.045 (0.006)
PC ae C42:2	Excluded	99.3	31.5	0.006 (0.003)	99.0	26.6	0.006 (0.002)
PC ae C42:3	Excluded	95.6	27.9	0.005 (0.003)	96.6	27.2	0.005 (0.002)
PC ae C42:4	Excluded	99.9	.	0 (0)	100.0	.	0.000 (0.000)
PC ae C42:5	Excluded	100.0	4.8	0.256 (0.014)	100.0	2.5	0.393 (0.019)
PC ae C44:3	Excluded	100.0	23.1	0.013 (0.003)	100.0	23.0	0.013 (0.003)
PC ae C44:4	Excluded	100.0	11.2	0.038 (0.005)	100.0	9.9	0.037 (0.004)
PC ae C44:5	Excluded	100.0	20.6	0.024 (0.006)	100.0	27.9	0.022 (0.006)
PC ae C44:6	Excluded	100.0	17.7	0.02 (0.004)	100.0	18.6	0.020 (0.004)
PC a C14:0	Excluded	100.0	6.0	1.542 (0.124)	100.0	4.3	1.993 (0.143)
PC a C16:0	Excluded	99.8	58.4	0.036 (0.049)	99.0	22.0	0.075 (0.245)
PC a C16:1	Excluded	97.7	26.1	0.028 (0.01)	95.1	22.0	0.035 (0.016)
PC a C17:0	Excluded	70.8	33.1	0.023 (0.012)	54.6	30.8	0.026 (0.014)
PC a C18:0	Excluded	99.9	26.8	0.082 (0.037)	99.0	10.7	0.098 (0.162)
PC a C18:1	Excluded	94.7	44.3	0.027 (0.024)	83.9	23.2	0.031 (0.071)
PC a C18:2	Excluded	83.5	54.6	0.023 (0.016)	82.9	42.1	0.034 (0.058)
PC a C20:3	Excluded	99.0	21.2	0.053 (0.015)	100.0	12.3	0.095 (0.020)
PC a C20:4	Excluded	54.5	41.0	0.012 (0.01)	28.8	49.3	0.015 (0.021)
PC a C24:0	Excluded	100.0	17.6	0.076 (0.015)	100.0	6.3	0.276 (0.028)
PC a C26:0	Excluded	96.7	41.3	0.012 (0.007)	86.8	35	0.011 (0.006)
PC a C26:1	Excluded	87.9	35.3	0.011 (0.006)	87.3	32.6	0.013 (0.007)
PC a C28:0	Excluded	100.0	19.9	0.056 (0.013)	100.0	14.1	0.059 (0.013)
PC a C28:1	Excluded	100.0	34.1	0.022 (0.016)	100.0	40	0.029 (0.018)

PC a C6:0	Excluded	20.0	27.3	0.03 (0.018)	12.7	19.8	0.049 (0.029)
**Sphingomyelins**							
SM (OH) C14:1	Excluded	36.5	50.6	0.015 (0.011)	98.5	.	0.000 (0.004)
SM (OH) C16:1	Excluded	53.1	164.5	0.002 (0.004)	32.7	116.7	0.004 (0.008)
SM (OH) C22:1	Excluded	15.7	33.4	0.024 (0.02)	16.1	21.2	0.033 (0.037)
SM (OH) C22:2	Excluded	54.9	106.5	0.004 (0.007)	52.7	118.1	0.004 (0.013)
SM (OH) C24:1	Excluded	46.8	69.0	0.005 (0.005)	36.6	47.4	0.008 (0.008)
SM C16:0	Excluded	5.5	29.9	0.116 (0.139)	2.4	13.4	0.150 (0.272)
SM C16:1	Excluded	42.8	39.3	0.01 (0.011)	13.2	33.4	0.014 (0.026)
SM C18:0	Excluded	16.0	47.7	0.019 (0.02)	14.6	23.3	0.026 (0.043)
SM C18:1	Excluded	44.7	67.3	0.004 (0.007)	42.9	49.7	0.006 (0.017)
SM C20:2	Excluded	84.3	150.9	0.001 (0.002)	70.7	258.2	0.001 (0.002)
SM C22:3	Excluded	69.4	88.8	0.003 (0.003)	75.1	80.8	0.003 (0.003)
SM C24:0	Used	4.0	24.8	0.057 (0.048)	2.4	21.7	0.078 (0.094)
SM C24:1	Excluded	7.8	34.6	0.046 (0.055)	1.0	23.0	0.066 (0.108)
SM C26:0	Excluded	70.0	61.5	0.006 (0.011)	28.8	89.9	0.009 (0.016)
SM C26:1	Excluded	91.0	75.1	0.003 (0.004)	76.6	67.8	0.004 (0.006)
**Hexoses**							
H1	Used	0.1	16.1	823.026 (961.181)	2.0	8.0	1344.560 (5852.640)

Table S2. Results of 15 metabolites significantly altered by night shift work in combined and chronotype-stratified analysis based on creatinine-normalized values in the basic model

Shown are the results for the β-estimate, the 95% confidence interval (CI) and false discovery rate (FDR) of the basic LMEM for the comparison of day shift (reference) and night. The basic model was adjusted for chronotype and batch effect; Significant *P*-values (FDR < 0.05) in both the basic and full LMEM model are indicated in bold. N = Number of nurses; n = Number of samples.

Metabolites	Combined analysis N = 68; n = 424		Early chronotype N = 16; n=91		Intermediate chronotype N = 22; n = 141		Late chronotype N = 30; n = 192	
	β–Estimate (95% CI)	FDR p-value	β–Estimate (95% CI)	FDR p-value	β-Estimate (95% CI)	FDR p-value	β-Estimate (95% CI)	FDR p-value
C5	0.07 (0.01, 0.12)	**5.0E-2**	0.22 (0.10, 0.35)	**2.6E-3**	-0.03 (-0.13, 0.07)	8.2E-1	0.09 (0.00, 0.17)	1.8E-1
C7-DC	0.20 (0.09, 0.31)	**2.3E-3**	0.55 (0.30, 0.80)	**7.8E-4**	0.05 (-0.15, 0.25)	8.7E-1	0.14 (-0.02, 0.29)	2.6E-1
C8	0.13 (0.03, 0.23)	**2.9E-2**	0.50 (0.27, 0.74)	**8.1E-4**	-0.07 (-0.26, 0.12)	7.6E-1	0.14 (0.00, 0.27)	1.8E-1
C10	0.16 (0.06, 0.26)	**8.9E-3**	0.51 (0.25, 0.78)	**1.3E-3**	-0.01 (-0.19, 0.18)	9.6E-1	0.14 (0.01, 0.26)	1.5E-1
C10:2	-0.38 (-0.53, -0.22)	**3.1E-5**	-0.35 (-0.73, 0.04)	1.4E-1	-0.21 (-0.44, 0.01)	3.8E-1	-0.45 (-0.68, -0.21)	**3.7E-3**
C12	0.26 (0.15, 0.38)	**7.1E-5**	0.62 (0.35, 0.90)	**7.8E-4**	0.11 (-0.12, 0.34)	7.1E-1	0.21 (0.06, 0.35)	**4.9E-2**
C14	0.14 (0.03, 0.24)	**4.2E-2**	0.47 (0.18, 0.76)	**5.6E-3**	0.04 (-0.17, 0.24)	8.9E-1	0.05 (-0.09, 0.18)	7.8E-1
C14:1	0.16 (0.06, 0.26)	**8.9E-3**	0.51 (0.26, 0.76)	**8.1E-4**	0.10 (-0.09, 0.28)	7.1E-1	0.03 (-0.09, 0.16)	8.2E-1
C14:1-OH	0.18 (0.07, 0.30)	**8.9E-3**	0.55 (0.26, 0.85)	**1.8E-3**	0.11 (-0.11, 0.33)	7.1E-1	0.05 (-0.09, 0.19)	7.8E-1
C14:2	0.16 (0.06, 0.26)	**1.1E-2**	0.48 (0.20, 0.76)	**3.5E-3**	0.07 (-0.12, 0.26)	7.6E-1	0.08 (-0.05, 0.21)	5.4E-1
C14:2-OH	0.15 (0.04, 0.26)	**2.6E-2**	0.50 (0.21, 0.79)	**3.5E-3**	0.05 (-0.16, 0.25)	8.7E-1	0.03 (-0.10, 0.17)	8.2E-1
Gly	0.15 (0.04, 0.25)	**2.6E-2**	-0.13 (-0.36, 0.10)	3.9E-1	0.22 (0.04, 0.40)	1.6E-1	0.21 (0.04, 0.38)	9.5E-2
Phe	0.34 (0.21, 0.46)	**5.9E-6**	0.35 (0.04, 0.66)	5.9E-2	0.37 (0.14, 0.59)	6.5E-2	0.34 (0.16, 0.51)	**3.7E-3**
Ser	0.16 (0.04, 0.27)	**2.6E-2**	0.02 (-0.22, 0.27)	8.7E-1	0.21 (0.02, 0.40)	2.7E-1	0.18 (0.00, 0.36)	1.8E-1
SM C24:0	0.20 (0.12, 0.29)	**4.3E-5**	0.42 (0.19, 0.66)	**2.6E-3**	0.12 (-0.05, 0.28)	5.1E-1	0.19 (0.09, 0.29)	**3.7E-3**
